# Author Correction: In silico comparison of SARS-CoV-2 spike protein-ACE2 binding affinities across species and implications for virus origin

**DOI:** 10.1038/s41598-021-98366-1

**Published:** 2021-09-14

**Authors:** Sakshi Piplani, Puneet Kumar Singh, David A. Winkler, Nikolai Petrovsky

**Affiliations:** 1 College of Medicine and Public Health, Flinders University, Bedford Park, 5046 Australia; 2 Vaxine Pty Ltd, 11 Walkley Avenue, Warradale, 5046 Australia; 3 Department of Biochemistry and Genetics, La Trobe Institute for Molecular Science, La Trobe University, Melbourne, VIC 3086 Australia; 4 Monash Institute of Pharmaceutical Sciences, Monash University, Parkville, 3052 Australia; 5 School of Pharmacy, University of Nottingham, Nottingham, NG7 2RD UK

Correction to: *Scientific Reports* 10.1038/s41598-021-92388-5, published online 24 June 2021

The original version of this Article contained an error in discussing the work of Rodrigues and colleagues. The text in the Results and discussion section, under the subheading ‘Comparisons of our model predictions to other studies’,

“While this paper was being prepared, Rodrigues et al. published a study that used the HADDOCK docking method to estimate the relative strength of binding affinities of SARS-CoV-2 spike protein for ACE2 proteins of 30 species^52^, with the docking followed by very short MD simulations. That study similarly generated results that do not accord with experimental data, with claims that 18 species of ACE2 had higher affinity for SARS-Cov-2 spike protein than human ACE2, including dog, pangolin, ferret, Siberian tiger, horseshoe bat, civet, hamster, guinea pig, goldfish, sheep, cat, horse and rabbit. Goldfish ACE2 was claimed to have the second highest affinity for S protein after dog ACE2, despite fish having no known permissiveness for SARS-Cov-2 infection. These erroneous results from simple docking studies indicate that more sophisticated and rigorous computational methods, such as our own, are required to obtain binding affinities consistent with experimental observations^52^. Indeed those authors admitted that their predictions were not correct and, for example, they ranked guinea pig ACE2 (non-susceptible species) as having higher binding for S protein than human, cat, horse, or rabbit (all susceptible species) contradicting experimental data showing that there is negligible binding of S protein with guinea pig ACE2^53^. The reasons for poor results of these other modelling studies may be that MD simulations were very short or absent. Notably, much longer MD simulations like those used in our current study are needed to obtain accurate ranking of binding affinities.”

now reads:

“Rodrigues et al. published a study that used the HADDOCK docking method to estimate the relative strength of binding affinities of SARS-CoV-2 spike protein for ACE2 proteins of 30 species^52^ with the docking including short restrained MD simulations. As noted by the authors, computational models have limitations, requiring validation against experimental data. For example, their model scored guinea pig and goldfish ACE2 among susceptible species, though it has been shown that guinea pig is non-susceptible^53^, and indirect evidence suggests fish to be naturally resistant. This suggests that longer and potentially more accurate simulation protocols such as the one used in our work, are required to obtain sufficiently precise results to allow accurate comparison of differences in calculated binding energies.”

The original Article has been corrected.

